# The expression level of *BAALC*-associated microRNA miR-3151 is an independent prognostic factor in younger patients with cytogenetic intermediate-risk acute myeloid leukemia

**DOI:** 10.1038/bcj.2015.76

**Published:** 2015-10-02

**Authors:** M Díaz-Beyá, S Brunet, J Nomdedéu, A Cordeiro, M Tormo, L Escoda, J M Ribera, M Arnan, I Heras, D Gallardo, J Bargay, M P Queipo de Llano, O Salamero, J M Martí, A Sampol, C Pedro, M Hoyos, M Pratcorona, J J Castellano, M Nomdedeu, R M Risueño, J Sierra, M Monzó, A Navarro, J Esteve

**Affiliations:** 1 Hematology Department, IDIBAPS, Hospital Clinic, Barcelona, Spain; 2 Josep Carreras Leukaemia Research Institute, Barcelona, Spain; 3 Hematology Department and Biological Hematology Laboratory, Hospital de Sant Pau, Barcelona, IIB-Sant Pau Research Institute, Universitat Autonoma of Barcelona, Barcelona, Spain; 4 Molecular Oncology and Embryology Laboratory, Human Anatomy Unit, School of Medicine, University of Barcelona, Barcelona, Spain; 5 Hematology Department, Hospital Clínico, Valencia, Spain; 6 Hematology Department, Hospital Joan XXIII, Tarragona, Spain; 7 Hematology Department, Institut Català d'Oncologia (ICO)-Hospital Germans Trias i Pujol, Badalona, Spain; 8 ICO, Hematology Department, Hospital Duran i Reynals, l'Hospitalet de Llobregat, Barcelona, Spain; 9 Hematology Department, Hospital Morales Meseguer, Murcia, Spain; 10 Hematology Department, ICO Josep Trueta, Girona, Spain; 11 Hematology Department, Hospital de Son Llàtzer, Palma de Mallorca Hematology, Palma de Mallorca, Spain; 12 Hematology Department, Hospital Virgen de la Victoria, Malaga, Spain; 13 Hematology Department, Hospital Vall d'Hebron, Barcelona, Spain; 14 Hematology Department, Hospital Mutua de Terrassa, Barcelona, Spain; 15 Hematology Department, Hospital de Son Llàtzer, Palma of Mallorca, Spain; 16 Hematology Department, Hospital de Mar, Barcelona, Spain; 17 University of Barcelona, Barcelona, Spain

## Abstract

Acute myeloid leukemia (AML) is a heterogeneous disease whose prognosis is mainly related to the biological risk conferred by cytogenetics and molecular profiling. In elderly patients (⩾60 years) with normal karyotype AML miR-3151 have been identified as a prognostic factor. However, miR-3151 prognostic value has not been examined in younger AML patients. In the present work, we have studied miR-3151 alone and in combination with *BAALC*, its host gene, in a cohort of 181 younger intermediate-risk AML (IR-AML) patients. Patients with higher expression of miR-3151 had shorter overall survival (*P*=0.0025), shorter leukemia-free survival (*P*=0.026) and higher cumulative incidence of relapse (*P*=0.082). Moreover, in the multivariate analysis miR-3151 emerged as independent prognostic marker in both the overall series and within the unfavorable molecular prognostic category. Interestingly, the combined determination of both miR-3151 and *BAALC* improved this prognostic stratification, with patients with low levels of both parameters showing a better outcome compared with those patients harboring increased levels of one or both markers (*P*=0.003). In addition, we studied the microRNA expression profile associated with miR-3151 identifying a six-microRNA signature. In conclusion, the analysis of miR-3151 and *BAALC* expression may well contribute to an improved prognostic stratification of younger patients with IR-AML.

## Introduction

The biological heterogeneity of acute myeloid leukemia (AML) results in a markedly diverse prognosis and highly differential sensitivity to current standard therapy. The most accurate prognostic stratification is based on cytogenetics, further refined with the analysis of several gene mutations.^1, 2, 3, 4^ In cytogenetic intermediate-risk AML (IR-AML), comprising ~50% of all AML patients, the mutational status of *NPM1*, internal tandem duplication of *FLT3* (FLT3-ITD), and *CEBPA* defines molecular subcategories with different biological risk and diverse outcomes.^2, 4^ Therefore, many current AML treatment protocols adapt their therapeutic algorithm to this biological risk. Nonetheless, despite several attempts to fine-tune stratification based on these molecular subcategories, the prognosis of many patients with IR-AML is still uncertain and the optimal post-remission therapy is unclear.

Several studies have shown the importance of microRNA (miRNA) deregulation in AML.^5, 6, 7^ Distinctive miRNA profiles have been associated with specific cytogenetic subtypes: AML associated with translocation t(8;21), t(15;17) or inv(16) and *MLL*-rearranged AML;^8, 9^ t(8;16) AML;^10^ and AML with specific gene mutations,^11^ including *NPM1*,^9, 12^ *FLT3*^9, 13^ and *CEBPA*.^8, 9, 10, 13, 14, 15^ In addition, several miRNAs have been associated with clinical outcome.^11^ Three studies that included cytogenetically heterogeneous AML cohorts found that expression levels of miR-191 and miR-199a,^13^ miR-196b^16^ and miR-212^(ref. 16)^ were associated with overall survival (OS). In patients with cytogenetically normal AML (CN-AML) and high-risk molecular features, a 12-miRNA prognostic signature was proposed by the CALGB group; this signature included five members of the miR-181 family.^13^ The individual prognostic value of miR-181a was later confirmed in a cohort of CN-AML patients by the same group.^17^ miR-155 has also been described as a prognostic marker in both older and younger CN-AML patients by the CALGB group.^18^ Our group has reported four miRNAs (miR-196b, miR-644, miR-135a and miR-409-3p) with prognostic value in IR-AML.^19^

miR-3151, which was first identified by massive sequencing techniques,^20, 21^ is located within the first intron of the *BAALC* gene. In a CALGB study including only CN-AML patients older than 60 years, miR-3151 was identified as an independent prognostic factor.^22^ In the same study, patients with overexpression of both miR-3151 and *BAALC* had the worst outcome, while those with low levels of both had the best outcome. Moreover, the authors described an mRNA/miRNA profile associated with higher miR-3151 expression. The same group later reported that miR-3151 and *BAALC* were both regulated by SP1/NF-KB, while *BAALC* expression (but not miR-3151) was regulated by the transcription factor RUNX1. Moreover, TP53 was identified as a target of miR-3151, and higher levels of miR-3151 induced leukemogenesis in a murine model and reduced apoptosis and chemosensitivity in AML cell lines.^23^

Although the CALGB study included only older patients with CN-AML,^22^ grouping patients with CN-AML together with those with other cytogenetic intermediate-risk alterations in the same prognostic category seems to be warranted from the clinical standpoint.^2^ Furthermore, younger patients have diverse options for post-remission strategies, including the possibility of an allogeneic hematopoietic stem cell transplantation, depending on biological risk, highlighting a need for prognostic markers in this group. Nevertheless, the prognostic value of miR-3151 expression has not been explored in younger IR-AML patients. We have examined the effect of miR-3151 expression in 181 patients with all types of cytogenetic IR-AML and correlated our findings with outcome.

## Subjects and methods

### Patients and treatment

We selected patients with untreated, *de novo* IR-AML according to the MRC classification^3^ and with available RNA samples at diagnosis for miRNA analysis (Table 1). All patients provided their written informed consent in accordance with the Declaration of Helsinki, and the Ethics Committee of Hospital Clinic of Barcelona approved the study.

All 181 IR-AML patients were treated from 1994 to 2009 in any of 16 centers participating in three consecutive trials of intensive chemotherapy for fit patients of the Spanish AML cooperative group CETLAM: AML-94 (*n*=9); AML-99 (NCT01716793) (*n*=26); AML-03 (NCT01723657) (*n*=146). Briefly, the induction regimen of AML-94 was ICE (idarubicin, standard-dose cytarabine and etoposide), while in AML-99 and AML-03 it consisted of one or two courses of IDICE (idarubicin, intermediate-dose cytarabine and VP-16), with or without priming with G-CSF, respectively. All patients achieving complete remission (CR) received an additional course of chemotherapy with mitoxantrone and high-dose cytarabine, and then a transplant decision was made. In protocols AML-99 and AML-03, an autologous HSCT was planned for patients harboring a normal karyotype without additional risk factors, whereas alloHSCT in first CR (CR1) was recommended for the remaining patients with an available donor. Risk factors considered for risk assignment were the need for two induction courses to achieve CR, detectable minimal residual disease by flow cytometry after intensification therapy (AML-03), and presence of *FLT3*-ITD (AML-03). In AML-94, post-remission strategy (autoHSCT vs alloHSCT) depended exclusively on the availability of an HLA-identical sibling.

### Molecular analysis

*NPM1* and *FLT3*-ITD mutations were assessed on genomic DNA as previously described^24, 25^ with labeled primers and analyzed by fragment analysis (3130XL Genetic Analyzer, Applied Biosystems (AB), Foster City, CA, USA). *CEBPA* mutations were analyzed as previously described.^14, 26^

### RNA extraction

Samples were obtained from bone marrow aspirates in 171 (94%) patients, and from peripheral blood, with a minimum blast infiltration of 80%, in the remaining 10 patients. Mononuclear cells were purified by Ficoll density gradient centrifugation and total RNA was isolated using Trizol reagent according to manufacturer's protocol (Invitrogen, Paisley, UK). All patients provided their written informed consent in accordance with the Declaration of Helsinki, and the Ethics Committee of each participating institution approved the study.

### miRNA quantification

The expression of miR-3151 was analyzed using TaqMan MicroRNA Assay (Applied Biosystems 243597_mat). Ten nanograms of total RNA were used for miRNA quantification. TaqMan microRNA assays (AB) for miR-3151 were used as previously described^27^ in an AB 7500 Sequence Detection System. Relative quantification was calculated using 2^-ΔΔCt^. Normalization was performed with RNU48 (Applied Biosystems 4427975). All experiments were performed in triplicate.

We had previously performed a comprehensive miRNA expression analysis, comprising 670 mature human miRNAs, in tumor samples from the 78 out of the 181 patients^10^ using TaqMan Array Human MicroRNA Set Cards v2.0 (AB).

### mRNA expression analysis

cDNA was synthesized from 1000 ng of total RNA using TaqMan Reverse Transcription Reagent Kit (Applied Biosystems). TaqMan Gene expression assays (Applied Biosystems) were used to determine mRNA levels of *BAALC* (Hs00227249_m1) and *GUSB* (Hs00939627_m1), used as housekeeping gene. Real-time PCR was performed in the ABI Prism 7500 Sequence Detection System (Applied Biosystems). All samples for each gene were run in triplicate and relative quantification was calculated using 2^−ΔΔCt^. Normalization was performed with *GUSB*.

### Molecularly defined prognostic subgroups in IR-AML

The presence or absence of *FLT3*-ITD, *NPM1* and biallelic *CEBPA* mutations have a strong prognostic impact in patients with IR-AML.^2, 4^ According to the European LeukemiaNet prognostic classification, patients with the *NPM1* mutation or the biallelic *CEBPA* mutation but without the *FLT3*-ITD mutation, when associated to normal cytogenetics, comprise a favorable genetic group—with better prognosis—while patients with the *FLT3*-ITD mutation and/or without the *NPM1* and the biallelic *CEBPA* mutation comprise the intermediate-I and intermediate-II genetic groups. In the present study, we have classified all IR-AML patients with *NPM1* mutations or biallelic *CEBPA* mutations but without *FLT3*-ITD mutations as the favorable molecular (FAVmol) subgroup and all remaining IR-AML patients as the unfavorable molecular (UNFAVmol) subgroup.

### Clinical endpoints and statistical methods

Expression levels of miR-3151 and *BAALC* were correlated with patient outcome. OS was calculated from diagnosis to death or last follow-up and leukemia-free survival (LFS) from CR to relapse or death. Both OS and LFS were estimated with the Kaplan–Meier method and comparisons among subgroups of patients were performed using the log-rank test. Relapse risk was calculated from CR to relapse and estimated using the cumulative incidence of relapse (CIR) method computed with the cmprsk package for R 2.12 software (The R Foundation for Statistical Computing, http://www.R-project.org/). The competing event in the relapse risk analysis was death without relapse. Comparison of relapse risk between groups of patients was performed using the Gray test.^28^ Characteristics between groups were compared using the *χ*^2^-test and Fisher's exact test, when applicable, for categorical variables, and the *t*-test for continuous variables. Multivariate analyses for OS and LFS were performed using the Cox proportional hazards model including age (10-year intervals), gender, white blood cell count (WBC; 50 × 10^9^/l increments) at diagnosis, mutational status of *NPM1* and *FLT3*-ITD, and miR-3151 and/or *BAALC* expression level. A multivariate analysis for CIR was performed using the subdistribution regression model of Fine and Gray^29^ with the cmprsk package. The proportional hazard assumption was tested for each variable by analyzing the Schoenfeld residuals. Kaplan–Meier survival curves were then drawn for miR-3151 and *BAALC* expression predicted to show a survival risk either above or below average risk, using the cut-off points of miR-3151 and *BAALC* expression levels identified by MaxStat package of R software (The R Foundation for Statistical Computing, http://www.R-project.org/). All analyses were performed with SPSS v.20 (Chicago, IL, USA) or R software version 2.12.2 (The R Foundation for Statistical Computing, http://www.R-project.org/). Significance was set at ⩽0.05.

To identify miRNAs with significant differential expression correlated with miR-3151 expression, data obtained from our previously identified miRNA profile^10^ were analyzed using BRB Array Tools version 3.5.0 software (Richard Simon & BRB-ArrayTools Development Team, http://linus.nci.nih.gov/BRB-ArrayTools.html, National Cancer Institute, Bethesda, MD, USA) and TIGR Multiexperiment viewer version 4.0 software (The Institute for Genomic Research, and ArrayAssist software, Stratagene, http://www.tm4.org/mev; Boston, MA, USA). A student's *t*-test based on multivariate permutation was performed with adjustment for multiple comparisons and with random variance model. Differences between miRNAs were considered statistically significant if the *P*-value was <0.01.

## Results

### miR-3151 expression and clinical and molecular characteristics

**Figure 1:**
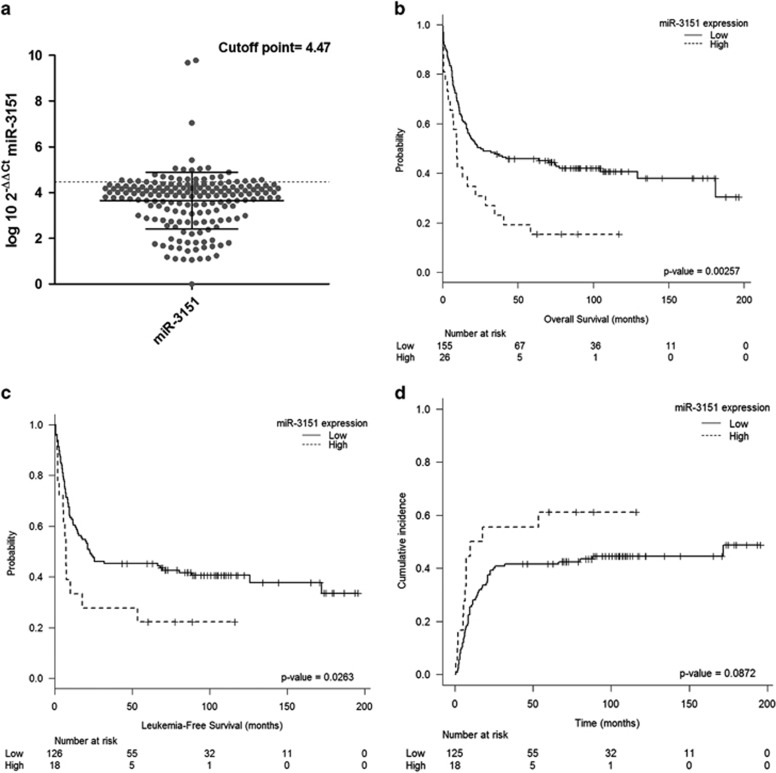
miR-3151 and outcome in younger IR-AML patients. (**a**) The optimal cut-off level for miR-3151 expression as identified by the MaxStat package. (**b**) Overall survival according to miR-3151 expression levels. (**c**) Leukemia-free survival according to miR-3151 expression levels. (**d**) Cumulative incidence of relapse according to miR-3151 expression levels.

miR-3151 was expressed with a variable expression level in the group of AML samples analyzed, with a median expression level of 3.94 (0–9.78) (Figure 1). The expression of miR-3151 was not significantly associated with any particular clinical feature, including age, WBC, bone marrow blast proportion, or FAB subtype, with the exception of *NPM1* mutation. Thus, miR-3151 was more expressed among patients without *NPM1* mutation and, using the same cut-off level as identified by the MaxStat package for prognostic purposes, patients with higher miR-3151 were more likely to harbor a wild-type *NPM1* configuration (80 vs 20%, *P*=0.009).

### miR-3151 expression has independent prognostic value in younger IR-AML patients

With a median follow-up of 8.4 years (range: 36-196 months) among patients alive at last follow-up, the 181 IR-AML patients had a CR rate of 83% and a 5-year OS, LFS and CIR of 42±7, 42±8 and 45±8%, respectively. Figure 1a shows the optimal cut-off level for miR-3151 expression as identified by the MaxStat package. Patients with higher miR-3151 levels experienced a worse outcome, with poorer OS and LFS (5-year OS: 15±13 vs 46±7%, *P*=0.0025; 5-year LFS: 22±18 vs 45±7%, *P*=0.026), and a higher CIR (71±20 vs 41.5±8%, *P*=0.082) compared with patients with lower levels (Figures 1b–d). In contrast, miR-3151 expression levels were not associated with the probability of attaining CR (83 vs 83%, *P*>0.99). Moreover, the proportion of patients who received an alloHSCT CR1 did not differ according to miR-3151 expression levels (*n*=4 (15%) vs *n*=38 (24%)) in patients with high and low miR-3151 levels, respectively; *P*=0.45).

A multivariate analysis confirmed high miR-3151 expression as an independent adverse prognostic factor for OS (OR: 2.97; 95% CI: 1.78–4.93; *P*<0.001) and LFS (OR: 2.65; 95% CI: 1.43–4.90; *P*=0.002) in addition to other variables with prognostic value such as age (OS, LFS), WBC count at diagnosis (OS), presence of *FLT3*-ITD (OS), and *NPM1* mutations (OS, LFS) (Table 2).

### Higher miR-3151 expression was associated with worse prognosis in both favorable and unfavorable molecular subgroups

**Figure 2:**
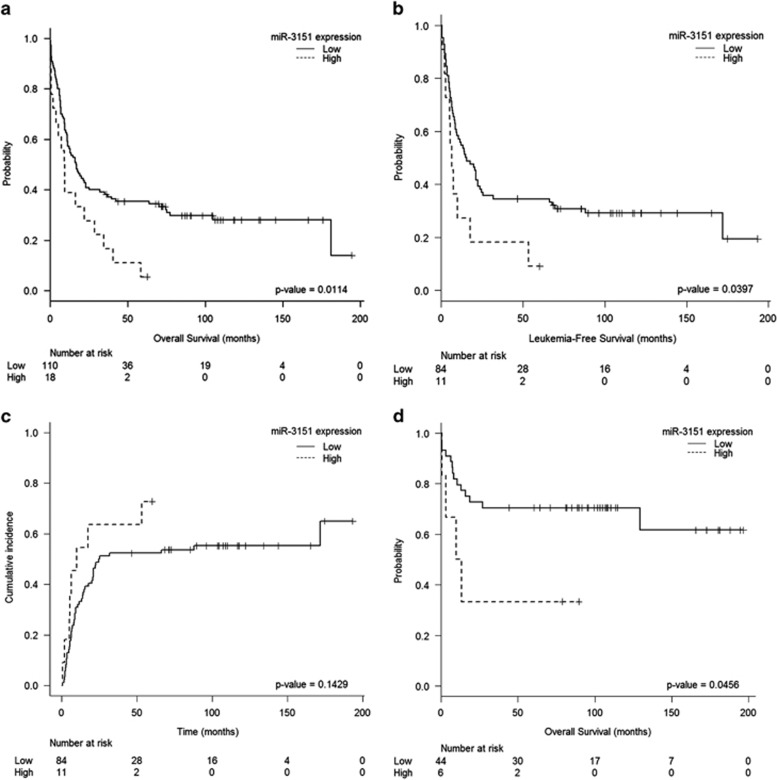
miR-3151 and outcome in younger IR-AML patients according to molecularly defined subgroups (FAVmol and UNFAVmol). (**a**) Overall survival according to miR-3151 expression levels in the UNFAVmol group. (**b**) leukemia-free survival according to miR-3151 expression levels in the UNFAVmol group. (**c**) cumulative incidence of relapse according to miR-3151 expression levels in the UNFAVmol group. (**d**) Overall survival according to miR-3151 expression levels in the FAVmol group.

We then analyzed the specific impact of miR-3151 expression on outcome in the molecularly defined subgroups (FAVmol and UNFAVmol). Among the 128 patients in the UNFAVmol subgroup, high miR-3151 expression identified a subset of patients with a very poor prognosis, with a shorter OS (5-year OS: 6±10 vs 35±8% *P*=0.011) and LFS (5-year LFS: 9±15 vs 34±10% *P*=0.04) and a trend towards higher CIR (*P*=0.1) (Figures 2a–c). In the multivariate analyses, miR-3151 expression retained its prognostic value in the UNFAVmol subgroup for OS (OR: 2.72; 95% CI: 1.53–4.84; *P*=0.001) and LFS (OR: 2.28; 95% CI: 1.10–4.72; *P*=0.026) (Table 2).

Despite the small size of the high miR-3151 expressers in the FAVmol subgroup (*n*=6 out of 50), high levels of miR-3151 were also associated with shorter OS (5-year OS: 33±38 vs 70±12% *P*=0.046) (Figure 2d), with a nonsignificant trend for LFS (*P*=0.1).

Given the different expression level according to *NPM1* mutation, prognostic impact was also analyzed separately in *NPM*1mut and *NPM1*wt cohorts. Thus, among *NPM1*wt AML patients, patients with higher miR-3151 levels showed a worse outcome, with a shorter OS (5-years OS: 5±10 vs 40±10% *P*=0.012) and LFS (5-year LFS: 7±14 vs 36±11% *P*=0.005) and a higher CIR (5-years CIR: 77±20 vs 52±12% *P*=0.033) (Supplementary Figures 1a–c).

### The combination of miR-3151 and *BAALC* expression provides additional independent prognostic value in IR-AML

**Figure 3:**
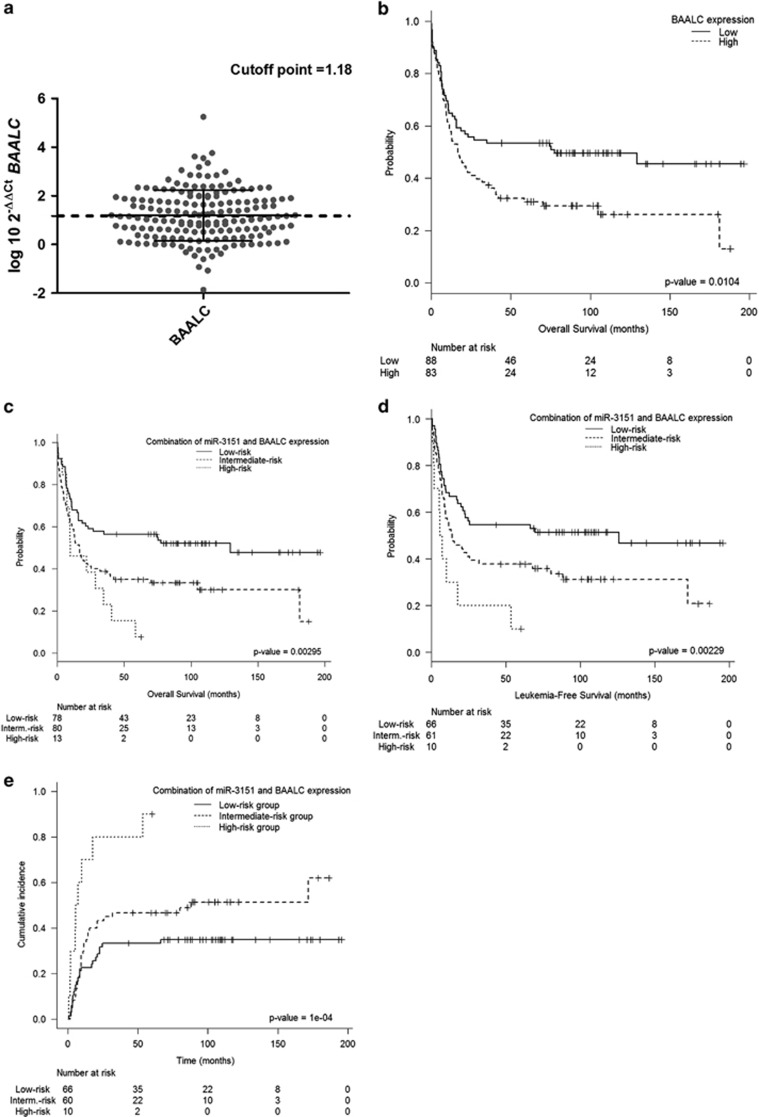
miR-3151 and *BAALC* expression and outcome in younger IR-AML patients. (**a**) The optimal cut-off level for *BAALC* expression as identified by the MaxStat package. (**b**) Overall survival according to *BAALC* expression levels. (**c**) Overall survival according to the combination of miR-3151 and *BAALC* expression levels. (**d**) leukemia-free survival according to the combination of miR-3151 and *BAALC* expression levels. (**e**) Cumulative incidence of relapse according to the combination of miR-3151 and *BAALC* expression levels.

In order to analyze the potential contribution to prognosis of the combined expression of miR-3151 and the expression of its host gene *BAALC*, we first studied the prognostic impact of *BAALC* expression in this cohort. The optimal cut-off point was determined using MaxStat (Figure 3a). Patients with higher *BAALC* expression had poorer OS than those with lower expression levels (5-year OS: 31±10 vs 50±10% *P*=0.01) (Figure 3b), poorer LFS (5-year LFS: 32±10 vs 54±10% *P*=0.004) and higher CIR (5-year CIR: 56±12 vs 32±12% *P*=0.0019).

Given the prognostic value shown by the expression level of miR-3151 and *BAALC* as individual markers in the present study and the prognostic value of both factors in combination demonstrated in a previous study performed by the CALGB study in older patients,^30^ we then analyzed the prognostic impact of the combination in our cohort of younger patients. High miR-3151 and high *BAALC* expression were defined as high-risk factors. Patients were classified into three groups according to the number of high-risk factors: low-risk group, 0 factors; intermediate-risk group, 1 factor; and high-risk group, 2 factors. For the low-, intermediate- and high-risk groups, 5-year OS was 56±10% 33±10% and 7±14%, respectively (*P*=0.003) (Figure 3c), 5-year LFS was 54±12%, 37±12%, and 1±18%, respectively (*P*=0.002) (Figure 3d), and 5-year CIR was 33±11%, 46±13%, and 90±37%, respectively (*P*<0.001) (Figure 3e). The multivariate analyses confirmed the combination as an independent prognostic factor in OS (OR: 1.65; 95% CI: 1.14–2.39; *P*=0.007), LFS (OR: 2.05; 95% CI: 1.26–3.32; *P*=0.004), and CIR (OR: 1.99; 95% CI: 1.19–3.39; *P*=0.008), after adjustment for other well-recognized molecular and clinical prognostic markers (Supplementary Table 1).

### Correlation of miR-3151 with miRNA expression

**Figure 4:**
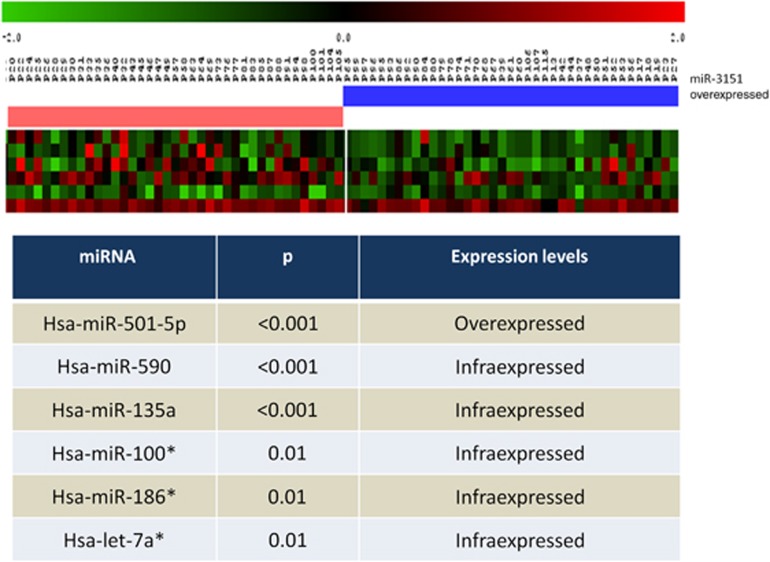
miRNA signature associated with high expression of miR-3151.

We had previously performed a comprehensive miRNA expression analysis in 78 patients of this cohort^10^ using TaqMan Array Human MicroRNA Set Cards v2.0 (AB). Remarkably, high expression of miR-3151 was associated with a miRNA signature in the group of patients with available miRNA profile, using BRB program from R. This signature comprised the overexpression of miR-501-5p (*P*<0.001), and downregulation of miR-590 (*P*<0.001), miR-135a (*P*<0.001), miR-100* (*P*=0.01), miR-186* (*P*=0.01) and let-7a* (*P*=0.01) (Figure 4).

## Discussion

miRNAs are involved in diverse essential functional pathways both in normal and neoplastic cells,^31, 32, 33^ and the expression of certain miRNAs in CN-AML or IR-AML has been shown to have prognostic relevance that can add useful information to molecular stratification based on the analysis of *NPM1*, *FLT3*-ITD and *CEBPA* mutations.^13, 19^ Here we have first demonstrated the independent prognostic value of miR-3151 expression and the combined determination of miR-3151 and its host gene in a series of younger IR-AML patients, both in the overall series and within molecularly defined subgroups with a differentiated prognosis. These results confirmed the prognostic impact of this miR-3151 previously observed in a AML cohort of older patients.^30^ Our results in a younger cohort might provide relevant clinical significance, by identification of a subgroup of IR-AML patients with a worse outcome who can be candidates to alloHSCT in an early phase. Moreover, miR-3151 can be particularly useful among patients without *NPM1* mutations, who lack an universal molecular marker for minimal residual disease monitoring as *NPM1*mut AML patients.

IR-AML, the largest AML cytogenetic subgroup, comprises patients with highly diverse prognosis, for whom the optimal therapeutic strategies still largely unclear in several subgroups. Currently prognostic stratification in these patients is based on the analysis of a limited number of molecular markers, mainly *NPM1*, *FLT3*-ITD and *CEBPA*, as recognized in the European LeukemiaNet risk classification.^1, 2, 3, 4^ Nonetheless the complex interaction of these markers with many other gene mutations that can modify the final prognostic impact, or the effect of intrinsic mutational characteristics, such as, allelic burden or mutation site on protein function and prognosis, make molecular risk stratification a highly complex process.^34^ In this context, the investigation of alternative biological AML features, such as gene or non-coding RNA expression, might provide relevant additional information on mechanisms of chemoresistance which might summarize the resultant effect of diverse combination of gene mutations. Few studies have identified several miRNA with prognostic value, including a 4-miRNA signature described by our group in the intermediate-risk AML cohort. miR-3151, described more recently, was not included in this previous study; its particular location, within a *BAALC* intron, a well-known AML prognostic factor,^35^ and its prognostic impact observed in an elderly AML cohort, prompted us to analyze its expression and prognostic effect in younger IR-AML patients.

For this prognostic evaluation, we included different IR-AML cytogenetic abnormalities beyond CN-AML, given the absence of outcome difference between both IR-AML cytogenetic subgroups (that is, normal an abnormal IR-AML karyotypes).^2^ Furthermore, IR-AML usually comprise all cytogenetic aberrations not allocated to good and poor-risk subgroups, and it is usually considered as the same risk category for post-remission therapy decision, which in younger patients includes the decision to perform an alloHSCT in CR1. Remarkably, miR-3151 expression analysis revealed prognostic value, with a detrimental effect among patients with higher levels. Of note, miR-3151 was especially informative in the subset of patients with an unfavorable genotype according to NPM1/FLT3-ITD/CEBPA configuration and in the group of patients lacking *NPM1* mutations, identifying a subgroup with a particular poor outcome. Availability of an additional prognostic tool in the poorest prognosis subsets could be of evident clinical interest in the subgroup of IR-AML patients not harboring a favorable genotype (such as those defined in the European LeukemiaNet favorable category). Moreover, prognostic value of miR-3151 value was independent of other well-characterized prognostic factors such as age, white blood cell count and gene mutations, and, interestingly, retained its prognostic value when our previously 4-miRNA score was included in the analysis (Supplementary Table 2). Prognostic impact of miR-3151 was even enriched with the simultaneous determination of *BAALC* which allowed us to build a simple score based on these two factors.

Importantly, although several miRNAs have been identified as prognostic markers in AML, their prognostic value has rarely been confirmed by subsequent studies by different cooperative groups. Here we have built on previous findings by CALGB showing the importance of miR-3151 and *BAALC* expression in older CN-AML patients^23^ and have validated their findings in younger IR-AML patients. In a further step, we have also identified a miRNA signature associated with higher expression of miR-3151, comprising a distinctive expression level of several miRNAs, such as let-7a*, miR-100*, miR-186*, miR-135a, miR-501-5p and miR-590.

We have shown in the present study that high expression of miR-3151—both alone and in combination with high *BAALC* expression—is an independent prognostic factor associated with poor outcome in younger IR-AML patients. These results confirmed for the first time the prognostic value of this miRNA previously observed in an older IR-AML cohort, and suggest that determination of its expression levels might improve risk stratification and post-remission treatment evaluation of younger IR-AML.

## Figures and Tables

**Table 1:** Main clinical characteristics of patients included in the study

*Intermediate risk AML n=181*	
Year of diagnosis (range)	1994–2009
*Gender n (%)*	
Male	97 (54%)
Female	84 (46%)
Median age, years (range)	51 (18–69)
Leukocyte count at diagnosis, × 10^9^/l median (range)	47 (1–408)

*FAB subtype (n)*	
M0	7
M1	53
M2	28
M4	48
M5	37
M6	6
M7	2

*Cytogenetics n (%)*	
Normal	131 (72%)
Other intermediate-risk	50 (28%)

*Molecular features n (%)*	
*NPM1* mutation	79 (43%)
*FLT3*-ITD	68 (37%)
*CEBPA* biallelic mutation	8 (7%)

*Therapeutic protocol (CETLAM group)*	
AML-94	9 (5%)
AML-99	26 (14%)
AML-03	146 (81%)

*Outcome*	
Complete response to induction regimen	83%
Overall survival (5- year)	42±7%
Leukemia-free survival (5- year)	42±8%
Cumulative incidence relapse (5- year)	45±8%
Allogeneic HSCT in first CR	42 (23%)

Abbreviations: AML, acute myeloid leukemia; CR, complete remission; HSCT, hematopoietic stem cell transplantation.

**Table 2:** Multivariate analyses for overall survival, leukemia-free survival and cumulative incidence of relapse in the overall series and in the molecularly defined UNFAVmol subgroup

*Variables*	P	*OR*	*95% CI*	P	*OR*	*95% CI*
	*All patients*			*UNFAVmol subgroup*		
*Overall survival*						
Age	<0.001	1.64	1.38–1.93	<0.001	1.60	1.32–1.91
Sex (male vs female)	0.28	1.24	0.83–1.84	0.18	1.35	0.87–2.10
WBC	0.006	1.21	1.06–1.38	0.08	1.14	0.98–1.32
*FLT3*-ITD	0.001	2.01	1.34–3.07	0.049	1.73	1.00–2.98
*NPM1* mutated	0.017	0.60	0.39–0.91	0.22	0.96	0.38–1.25
miR-3151 levels (high vs low)	<0.001	2.97	1.78–4.93	0.001	2.72	1.53–4.84

*Leukemia-free survival*						
Age	0.001	1.40	1.18–1.66	0.002	1.36	1.11–1.67
Sex (male vs female)	0.14	1.39	0.89–2.14	0.058	1.63	0.98–2.71
WBC	0.078	1.14	0.98–1.32	0.26	1.10	0.93–1.30
*FLT3*-ITD	0.081	1.53	0.94–2.46	0.706	1.14	0.56–2.32
*NPM1* mutated	0.02	0.58	0.36–0.91	0.35	0.69	0.32–1.50
miR-3151 levels (high vs low)	0.002	2.65	1.43–4.90	0.026	2.28	1.10–4.72

Abbreviations: CI, confidence interval; OR, odds ratio; UNFAVmol, unfavorable molecular group.

Age was analyzed with 10-year intervals and white blood cell count at diagnosis using 50x10^9^/l increments.

## References

[bib1] 1Grossmann V, Schnittger S, Kohlmann A, Eder C, Roller A, Dicker F et al. A novel hierarchical prognostic model of AML solely based on molecular mutations. Blood 2012; 120: 2963–2972. 22915647 10.1182/blood-2012-03-419622

[bib2] 2Döhner H, Estey EH, Amadori S, Appelbaum FR, Buchner T, Burnett AK et al. Diagnosis and management of acute myeloid leukemia in adults: recommendations from an international expert panel, on behalf of the European LeukemiaNet. Blood 2010; 115: 453–474. 19880497 10.1182/blood-2009-07-235358

[bib3] 3Grimwade D, Hills RK, Moorman AV, Walker H, Chatters S, Goldstone AH et al. Refinement of cytogenetic classification in acute myeloid leukemia: determination of prognostic significance of rare recurring chromosomal abnormalities among 5876 younger adult patients treated in the United Kingdom Medical Research Council trials. Blood 2010; 116: 354–365. 20385793 10.1182/blood-2009-11-254441

[bib4] 4Schlenk RF, Dohner K, Krauter J, Frohling S, Corbacioglu A, Bullinger L et al. Mutations and treatment outcome in cytogenetically normal acute myeloid leukemia. N Engl J Med. 2008; 358: 1909–1918. 18450602 10.1056/NEJMoa074306

[bib5] 5Chen J, Odenike O, Rowley JD. Leukaemogenesis: more than mutant genes. Nat Rev Cancer 2010; 10: 23–36. 20029422 10.1038/nrc2765 PMC2972637

[bib6] 6Garzon R, Liu S, Fabbri M, Liu Z, Heaphy CE, Callegari E et al. MicroRNA-29b induces global DNA hypomethylation and tumor suppressor gene reexpression in acute myeloid leukemia by targeting directly DNMT3A and 3B and indirectly DNMT1. Blood 2009; 113: 6411–6418. 19211935 10.1182/blood-2008-07-170589 PMC2710934

[bib7] 7Schotte D, Pieters R, Den Boer M. MicroRNAs in acute leukemia: from biological players to clinical contributors. Leukemia 2011; 26: 1–12. 21701489 10.1038/leu.2011.151

[bib8] 8Dixon-McIver A, East P, Mein CA, Cazier JB, Molloy G, Chaplin T et al. Distinctive patterns of microRNA expression associated with karyotype in acute myeloid leukaemia. PLoS One 2008; 3: e2141. 18478077 10.1371/journal.pone.0002141 PMC2373886

[bib9] 9Jongen-Lavrencic M, Sun SM, Dijkstra MK, Valk PJ, Lowenberg B. MicroRNA expression profiling in relation to the genetic heterogeneity of acute myeloid leukemia. Blood 2008; 111: 5078–5085. 18337557 10.1182/blood-2008-01-133355

[bib10] 10Diaz-Beya M, Navarro A, Ferrer G, Diaz T, Gel B, Camos M et al. Acute myeloid leukemia with translocation (8;16)(p11;p13) and MYST3-CREBBP rearrangement harbors a distinctive microRNA signature targeting RET proto-oncogene. Leukemia 2012; 27: 595–603. 23022987 10.1038/leu.2012.278

[bib11] 11Marcucci G, Mrozek K, Radmacher MD, Garzon R, Bloomfield CD. The prognostic and functional role of microRNAs in acute myeloid leukemia. Blood 2011; 117: 1121–1129. 21045193 10.1182/blood-2010-09-191312 PMC3056468

[bib12] 12Garzon R, Garofalo M, Martelli MP, Briesewitz R, Wang L, Fernandez-Cymering C et al. Distinctive microRNA signature of acute myeloid leukemia bearing cytoplasmic mutated nucleophosmin. Proc Natl Acad Sci USA 2008; 105: 3945–3950. 18308931 10.1073/pnas.0800135105 PMC2268779

[bib13] 13Marcucci G, Radmacher MD, Maharry K, Mrozek K, Ruppert AS, Paschka P et al. MicroRNA expression in cytogenetically normal acute myeloid leukemia. N Engl J Med 2008; 358: 1919–1928. 18450603 10.1056/NEJMoa074256

[bib14] 14Marcucci G, Maharry K, Radmacher MD, Mrozek K, Vukosavljevic T, Paschka P et al. Prognostic significance of, and gene and microRNA expression signatures associated with, CEBPA mutations in cytogenetically normal acute myeloid leukemia with high-risk molecular features: a Cancer and Leukemia Group B Study. J Clin Oncol. 2008; 26: 5078–5087. 18809607 10.1200/JCO.2008.17.5554 PMC2652095

[bib15] 15Li Z, Lu J, Sun M, Mi S, Zhang H, Luo RT et al. Distinct microRNA expression profiles in acute myeloid leukemia with common translocations. Proc Natl Acad Sci USA 2008; 105: 15535–15540. 18832181 10.1073/pnas.0808266105 PMC2563085

[bib16] 16Sun SM, Rockova V, Bullinger L, Dijkstra MK, Dohner H, Lowenberg B et al. The prognostic relevance of miR-212 expression with survival in cytogenetically and molecularly heterogeneous AML. Leukemia 2012; 27: 100–106. 22692398 10.1038/leu.2012.158

[bib17] 17Schwind S, Maharry K, Radmacher MD, Mrozek K, Holland KB, Margeson D et al. Prognostic significance of expression of a single microRNA, miR-181a, in cytogenetically normal acute myeloid leukemia: a Cancer and Leukemia Group B study. J Clin Oncol 2010; 28: 5257–5264. 21079133 10.1200/JCO.2010.29.2953 PMC3018359

[bib18] 18Marcucci G, Maharry KS, Metzeler KH, Volinia S, Wu Y-Z, Mrózek K et al. Clinical role of microRNAs in cytogenetically normal acute myeloid leukemia: miR-155 upregulation independently identifies high-risk patients. J Clin Oncol 2013; 31: 2086–2093. 23650424 10.1200/JCO.2012.45.6228 PMC3731981

[bib19] 19Díaz-Beyá M, Brunet S, Nomdedéu J, Tejero R, Díaz T, Pratcorona M et al. MicroRNA expression at diagnosis adds relevant prognostic information to molecular categorization in patients with intermediate-risk cytogenetic acute myeloid leukemia. Leukemia 2014; 28: 804–812. 24072101 10.1038/leu.2013.281

[bib20] 20Stark MS, Tyagi S, Nancarrow DJ, Boyle GM, Cook AL, Whiteman DC et al. Characterization of the melanoma miRNAome by deep sequencing. PLoS One 2010; 5: e9685. 20300190 10.1371/journal.pone.0009685 PMC2837346

[bib21] 21Schotte D, Akbari Moqadam F, Lange-Turenhout EA, Chen C, van Ijcken WF, Pieters R et al. Discovery of new microRNAs by small RNAome deep sequencing in childhood acute lymphoblastic leukemia. Leukemia 2011; 25: 1389–1399. 21606961 10.1038/leu.2011.105

[bib22] 22Eisfeld AK, Marcucci G, Maharry K, Schwind S, Radmacher MD, Nicolet D et al. miR-3151 interplays with its host gene BAALC and independently affects outcome of patients with cytogenetically normal acute myeloid leukemia. Blood 2012; 120: 249–258. 22529287 10.1182/blood-2012-02-408492 PMC3398762

[bib23] 23Eisfeld A-K, Schwind S, Patel R, Huang X, Santhanam R, Walker CJ et al. Intronic miR-3151 within BAALC drives leukemogenesis by deregulating the TP53 Pathway. Sci Signal 2014; 7: ra36. 24736457 10.1126/scisignal.2004762 PMC4165404

[bib24] 24Thiede C, Steudel C, Mohr B, Schaich M, Schakel U, Platzbecker U et al. Analysis of FLT3-activating mutations in 979 patients with acute myelogenous leukemia: association with FAB subtypes and identification of subgroups with poor prognosis. Blood 2002; 99: 4326–4335. 12036858 10.1182/blood.v99.12.4326

[bib25] 25Boissel N, Renneville A, Biggio V, Philippe N, Thomas X, Cayuela JM et al. Prevalence, clinical profile, and prognosis of NPM mutations in AML with normal karyotype. Blood 2005; 106: 3618–3620. 16046528 10.1182/blood-2005-05-2174

[bib26] 26Fröhling S, Schlenk RF, Stolze I, Bihlmayr J, Benner A, Kreitmeier S et al. CEBPA mutations in younger adults with acute myeloid leukemia and normal cytogenetics: prognostic relevance and analysis of cooperating mutations. J Clin Oncol 2004; 22: 624–633. 14726504 10.1200/JCO.2004.06.060

[bib27] 27Navarro A, Marrades RM, Vinolas N, Quera A, Agusti C, Huerta A et al. MicroRNAs expressed during lung cancer development are expressed in human pseudoglandular lung embryogenesis. Oncology 2009; 76: 162–169. 19209007 10.1159/000201569

[bib28] 28Gray RJ. A class of K-sample tests for comparing the cumulative incidence of a competing risk. Ann Stat 1988; 16: 1141–1154.

[bib29] 29Fine JP, Gray RJ. A proportional hazards model for the subdistribution of a competing risk. J Am Stat Assoc 1999; 94: 496–509.

[bib30] 30Eisfeld A-K, Marcucci G, Maharry K, Schwind S, Radmacher MD, Nicolet D et al. miR-3151 interplays with its host gene BAALC and independently affects outcome of patients with cytogenetically normal acute myeloid leukemia. Blood 2012; 120: 249–258. 22529287 10.1182/blood-2012-02-408492 PMC3398762

[bib31] 31Bartel DP. MicroRNAs: target recognition and regulatory functions. Cell 2009; 136: 215–233. 19167326 10.1016/j.cell.2009.01.002 PMC3794896

[bib32] 32Calin GA, Croce CM. MicroRNA signatures in human cancers. Nat Rev Cancer. 2006; 6: 857–866. 17060945 10.1038/nrc1997

[bib33] 33Rücker F, Russ A, Cocciardi S, Kett H, Schlenk R, Botzenhardt U et al. Altered miRNA and gene expression in acute myeloid leukemia with complex karyotype identify networks of prognostic relevance. Leukemia 2012; 27: 353–361. 22810507 10.1038/leu.2012.208

[bib34] 34Patel JP, Gönen M, Figueroa ME, Fernandez H, Sun Z, Racevskis J et al. Prognostic relevance of integrated genetic profiling in acute myeloid leukemia. N Engl J Med. 2012; 366: 1079–1089. 22417203 10.1056/NEJMoa1112304 PMC3545649

[bib35] 35Langer C, Radmacher MD, Ruppert AS, Whitman SP, Paschka P, Mrózek K et al. High BAALC expression associates with other molecular prognostic markers, poor outcome, and a distinct gene-expression signature in cytogenetically normal patients younger than 60 years with acute myeloid leukemia: a Cancer and Leukemia Group B (CALGB) study. Blood 2008; 111: 5371–5379. 18378853 10.1182/blood-2007-11-124958 PMC2396728

