# Supplementary figures and images for: The expression level of BAALC-associated microRNA miR-3151 is an independent prognostic factor in younger patients with cytogenetic intermediate-risk acute myeloid leukemia
Source: Blood Cancer J. 2015 Oct 2;5(10):e352–. doi: 10.1038/bcj.2015.76 (PMC4635188; doi:10.1038/bcj.2015.76)

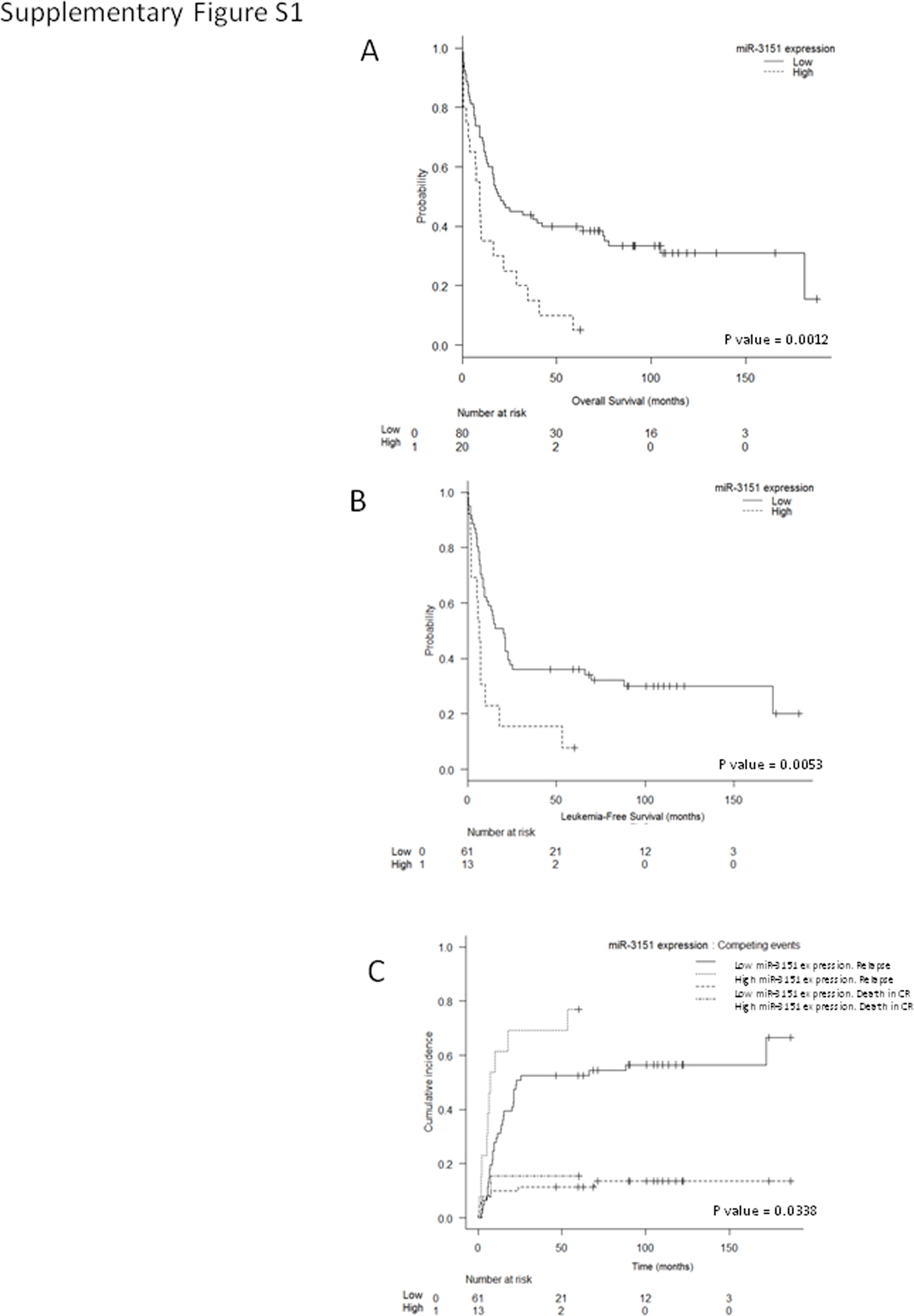

Supplement: Supplementary Figure S1 [file bcj201576x1.tif]
