# Supplementary material for: The expression level of BAALC-associated microRNA miR-3151 is an independent prognostic factor in younger patients with cytogenetic intermediate-risk acute myeloid leukemia
Source: Blood Cancer J. 2015 Oct 2;5(10):e352–. doi: 10.1038/bcj.2015.76 (PMC4635188; doi:10.1038/bcj.2015.76)
Supplement: Supplementary Figure Legends [file bcj201576x2.docx]

**Supplementary Figure 1. Outcome of IR-AML patients lacking *NPM1* mutation according to miR-3151 expression level at diagnosis: (a)** Overall survival; **(b)** Leukemia-free survival; **(c)** Cumulative incidence of relapse and death in complete remission in patients with low and high miR-3151 expression.
