# Supplementary material for: The expression level of BAALC-associated microRNA miR-3151 is an independent prognostic factor in younger patients with cytogenetic intermediate-risk acute myeloid leukemia
Source: Blood Cancer J. 2015 Oct 2;5(10):e352–. doi: 10.1038/bcj.2015.76 (PMC4635188; doi:10.1038/bcj.2015.76)
Supplement: Supplementary Tables [file bcj201576x3.docx]

**Supplementary Table 1.** Multivariate analyses for overall survival, leukemia-free survival, and cumulative incidence of relapse within the cytogenetic intermediate-risk cohort. Age was analyzed with 10-year intervals and white blood cell count at diagnosis using 50x10^9^/L increments.

| **Variables** | **p** | **OR** | **95%CI** |
| --- | --- | --- | --- |
|  | **Overall Survival** | | |
| **Age** | <0.001 | 1.64 | 1.38-1.93 |
| **Sex (male vs. female)** | 0.183 |  |  |
| **WBC** | 0.001 | 1.27 | 1.10-1.47 |
| ***FLT3*-ITD** | 0.011 | 1.753 | 1.13-2.71 |
| ***NPM1*  mutated** | 0.13 |  |  |
| **Combination miR-3151 and *BAALC* expression** | 0.007 | 1.65 | 1.15-2.40 |
|  | **Leukemia-free survival** | | |
| **Age** | 0.003 | 1.35 | 1.11-1.65 |
| **Sex (male vs. female)** | 0.48 |  |  |
| **WBC** | 0.031 | 1.22 | 1.02-1.471 |
| ***FLT3*-ITD** | 0.33 |  |  |
| ***NPM1* mutated** | 0.11 |  |  |
| **Combination miR-3151 and *BAALC* expression** | 0.004 | 2.05 | 1.26-3.33 |
|  | **Cumulative incidence of relapse** | | |
| **Age** | 0.014 | 1.28 | 1.05-1.55 |
| **Sex (male vs. female)** | 0.8 |  |  |
| **WBC** | 0.0086 | 1.24 | 1.05-1.461 |
| ***FLT3*-ITD** | 0.5 |  |  |
| ***NPM1*  mutated** | 0.09 |  |  |
| **Combination miR-3151 and *BAALC* expression** | 0.0085 | 1.99 | 1.19-3.33 |

**Supplementary Table 2.** Multivariate analyses for overall survival, within the cytogenetic intermediate-risk cohort. Age was analyzed with 10-year intervals and white blood cell count at diagnosis using 50x10^9^/L increments.

| **Variables** | **p** | **OR** | **95%CI** |
| --- | --- | --- | --- |
|  | **Overall Survival** | | |
| **Age** | <0.001 | 1.73 | 1.45-2.06 |
| **Sex (male vs. female)** | 0.66 |  |  |
| **WBC** | 0.16 | 1.18 | 1.03-1.35 |
| ***FLT3*-ITD** | 0.003 | 1.854 | 1.22-2.79 |
| ***NPM1*  mutated** | 0.013 | 0.586 | 0.385-0.892 |
| **Combination miR-3151 and *BAALC* expression** | <0.001 | 2.76 | 1.67-4.60 |
| **miR-score^19^** | 0.007 | 1.731 | 1.16-2.57 |
